# Profiling users and non-users of meal delivery services in Belgium using latent class analysis

**DOI:** 10.1186/s12966-025-01827-3

**Published:** 2025-10-30

**Authors:** Laura Helena Oostenbach, Matthew Keeble, Thomas Vanoutrive, Maartje P. Poelman, Carlijn B. M. Kamphuis, Wendy Van Lippevelde, Lukar Thornton

**Affiliations:** 1 Department of Marketing, Faculty of Business and Economics, University of Antwerp, Prinsstraat 13, Antwerp, 2000 Belgium; 2 Research Group for Urban Development, Faculty of Design Sciences & Urban Studies Institute, University of Antwerp, Mutsaardstraat 31, Antwerp, 2000 Belgium; 3 Department of Social Sciences, Chair Group Consumption & Healthy Lifestyles, Wageningen University & Research, Hollandseweg 1, Wageningen, 6706 KN The Netherlands; 4 Department of Interdisciplinary Social Science, Utrecht University, Padualaan 14, Utrecht, 3584 CH The Netherlands; 5 Department of Marketing, Innovation, and Organisation, Faculty of Economics and Business administration, Ghent University, Tweekerkenstraat 2, Ghent, 9000 Belgium

**Keywords:** Online food environment, Meal delivery services, Consumer behaviour, Latent class analysis, Belgium

## Abstract

**Background:**

Ready-to-eat meal delivery services have become increasingly popular in recent years, with potential detrimental health implications as the majority of food promoted and sold is energy-dense and nutrient-poor. However, limited research has examined who uses these services and why. This study explores the profiles of different users and non-users of meal delivery services and further describes the socio-demographic and health characteristics of these profiles.

**Methods:**

Data were from 1086 adults who completed the online 2024 Meal Delivery Survey conducted in the Flanders and Brussels regions of Belgium. Users of meal delivery services reported on usage indicators (e.g., valuing fast delivery, ordering to avoid traffic) and non-users reported on reasons for non-use (e.g., lacking trust in meal hygiene, finding it bad for the environment). Latent class analysis categorised users based on patterns of indicator endorsement. Amongst non-users, reasons for non-use were examined descriptively for both former users and never users. Socio-demographic and health characteristics were compared across profiles.

**Results:**

Over 66% of the sample (*n* = 720) were users and 34% (*n* = 366) non-users. Latent class analysis identified two user profiles. *Efficiency-focused users* (48% of user sample) solely prioritised fast and affordable service. *Variety- and convenience-driven users* (52% of user sample) endorsed a wider range of indicators, including the ability to try different cuisines, avoid supermarkets, and have more leisure time. The latter group included more frequent users. Amongst non-users, both *former users* (52% of non-user sample) and *never users* (48% of non-user sample) most commonly reported preferences for in-store food shopping and home cooking as reasons why they did not order meals for delivery. Profiles differed in socio-demographic and health characteristics. For example, *variety- and convenience-driven users* reported poorer health than *efficiency-focused users. Non-users (former and never)* were older than *users*, with *never users* having the oldest age profile.

**Conclusions:**

This study provides a better understanding of determinants of use and non-use of meal delivery services, informing public health action aimed at improving food behaviours and reducing the burden of diet-related diseases. Results can support the development of targeted interventions addressing main drivers of meal delivery service use.

**Supplementary Information:**

The online version contains supplementary material available at 10.1186/s12966-025-01827-3.

## Background

Ready-to-eat meal delivery services have become commonplace in recent years, with their growth accelerating during and after the COVID-19 pandemic [1, 2]. In Belgium, which has a population of almost 12 million [3], the number of users is expected to reach 4.6 million by 2029, generating a projected revenue of €717 million [4]. These services involve the delivery of meals prepared by out-of-home food outlets (e.g., restaurants, fast-food outlets) to customers who order through third-party meal delivery platforms (e.g., Uber Eats) or directly with restaurants via websites or phone. Such services provide access to meals for immediate consumption upon delivery. However, out-of-home meals are often more energy-dense and nutrient-poor than self-prepared meals [5]. International evidence suggests that the most commonly advertised meals for delivery tend to be unhealthy options such as pizzas and burgers [6–8]. Given the well-established links between unhealthy dietary patterns and chronic diseases (e.g., type 2 diabetes, and cardiovascular diseases) [9], and the increasing demand for meal delivery services [1, 4], it is important to understand both who uses these services and why. Further, healthy eating strategies can also be informed through an understanding of non-users and their motivations for non-use.

Whilst there is growing attention to meal delivery services in research and food policy [10–14], evidence on out-of-home food consumption predominantly focuses on the physical food environment, e.g., fast-food and takeaway outlets [15–17]. Prior work identified convenience, taste, and speed of consumption as main drivers of the consumption of such food [18, 19]. Widespread smartphone adoption and the rise of meal delivery services have likely further reinforced these drivers and made out-of-home food more readily available than ever before [20]. By removing the need for traditional physical interactions between consumers and food retailers, and relying on delivery drivers to bridge the distance, such services have expanded food environments and reduced the effort required to access out-of-home food [7]. Meal delivery services do not merely extend physical eat-in or takeaway options, they also include delivery-only restaurants (e.g., “dark kitchens”), which provide food exclusively for delivery without onsite options [21]. This shift in how consumers interact with food retailers may have impacted the profiles of out-of-home food users and factors motivating use. However, there has been scarcity of research focusing on meal delivery services compared to the brick-and-mortar food environment, with most existing research coming from a limited range of contexts, e.g., Australia and the United Kingdom [12, 14].

Most research on meal delivery services has emerged from the fields of marketing, business, hospitality, and transportation (e.g., [22–31]), mainly focusing on service quality and delivery application attributes (e.g., courier service quality, delivery time, real-time traceability of order location/status, and visual application design) as determinants of user satisfaction and usage. Recent public health research has started to explore user characteristics [32–34], with evidence suggesting users of meal delivery services are generally younger and more often live with overweight or obesity [32, 33]. However, findings related to the income and education levels of users are inconsistent [32–34], and factors motivating the use of meal delivery services remain largely unexplored. Similarly, little is known about the reasons for non-use. It remains unclear whether some individuals do not use these services due to lack of access in their area or whether other factors are at play.

This study aims to (1) identify profiles of users of ready-to-eat meal delivery services based on indicators related to meal delivery ordering, (2) explore reasons for non-use amongst individuals who do not use these services, and (3) characterise users and non-users in terms of their socio-demographic and health attributes. The study provides a better understanding of consumer behaviours related to meal delivery services in the Belgian context, where the use of these services is growing [4]. It is important to note that this study specifically focuses on the delivery of ready-to-eat meals and does not include the delivery of groceries, meal kits, or ready-to-heat meals.

## Methods

### Context

This study used data from participants (*n* = 1304) who completed the online 2024 Meal Delivery Survey as part of the Flemish “Fonds Wetenschappelijk Onderzoek” (FWO) funded project “Tracking the growth and impact of the ready-to-eat meal delivery system in Belgium”. The project received ethics approval from the University of Antwerp Ethics Committee for the Social Sciences and Humanities (ex_SHW_2023_32_1).

### Study design and participant recruitment

The cross-sectional online Meal Delivery Survey was conducted in the Flanders and Brussels regions of Belgium and administered in Qualtrics [35]. Many of the survey questions were adapted from previous surveys [36–38]. The survey included one common section for all respondents, capturing details about general topics, e.g., socio-demographic characteristics, and two separate sections; one for users, capturing factors related to meal delivery service use, and another for non-users, capturing their reasons for not using meal delivery services. This separated structure allowed us to ask specific questions related to use and non-use rather than more general questions. The survey was available in English, Dutch and French (the latter two being the official and most commonly spoken languages in the regions under study). Participant recruitment was conducted over an eleven-week period (16th April 2024–7th July 2024). Digital flyers inviting people to complete the online survey were also created in English, Dutch and French, and were shared through non-paid posts on various public Facebook groups and online communities on Reddit. They were also shared within the researchers’ own networks via LinkedIn, WhatsApp and X (formerly known as Twitter). To reach a broader audience, paid posts were advertised via Meta Ads across various platforms such as Facebook, Instagram, and Messenger from 3rd May 2024 to 7th July 2024. Online advertising, recruitment and survey delivery was a deliberate choice to ensure the sample were participants who had access to the internet and had a minimal level of digital literacy. This meant that for non-users the sample was essentially individuals with the technology that could allow them to order meals for delivery and, thus, allowed us to explore motivations for non-use beyond simply not having internet access or digital illiteracy. Eligible participants had to be aged 18 years or older and live in the Brussels or Flemish regions, in Belgium. Additional file 1 provides a flowchart of inclusion and exclusion of respondents.

### Sample characteristics

Socio-demographic characteristics included gender (male/female), age (continuous), ability to manage on income (very difficult or difficult/just getting by/comfortable or very comfortable), employment status (employed/studying/unemployed or homemaker or unable to work/retired), highest educational attainment (university/less than university), residential location type (city centre/outskirts/village centre/countryside or along a connecting road), living status (alone/with other people), and presence of children in the household (no children/at least one child aged 4 years or younger/only child(ren) aged over 4 years). Health characteristics included body mass index (BMI) calculated from self-reported weight (kilograms) and height (metres) (BMI = weight/height^2^) (continuous) and self-rated health (poor or fair/good/very good or excellent). The corresponding survey questions and response categories are detailed in Additional file 2.

### Use of meal delivery services

Two questions were used to distinguish between users and non-users. First, all respondents were asked if they ever had a ready-to-eat meal delivered to their home, for example, pizza or sushi ordered through online applications such as Uber Eats, Takeaway.com, or directly from a restaurant website, or via phone call to a restaurant. Second, those who said yes were further asked whether they had had a ready-to-eat meal delivered to their home in the last 6 months. This cut-off point was used to reflect recent food practices. Respondents who said yes to usage in the last 6 months were considered *users* of meal delivery services and received the questionnaire focusing on usage. Users were further asked to report how often they order a meal for delivery in a typical month (once or less than once per month/twice or more per month) and the methods they use when ordering (meal delivery applications, restaurant website, phone call), where they could indicate yes/no for each option (Additional file 2). Respondents who said yes to ever using but no to usage in the last 6 months were considered as currently being non-users, termed *former* users, and received the questionnaire focusing on reasons for non-use. This term aims to highlight that they had prior experience with these services but had not recently used them. Respondents who said no to ever using meal delivery services were considered non-users, more specifically *never* users, and received the questionnaire focusing on reasons for non-use.

### Indicators related to meal delivery ordering amongst users

Users reported on indicators related to their use of meal delivery services. The indicators included their meal ordering behaviours (e.g., order based on promotions), important factors when ordering (e.g., fast delivery), and reasons for ordering (e.g., limited/no cooking skills) (Table 2). All indicators were binary (yes/no) as the survey was designed with the intention of conducting future latent class analysis (LCA), a method that typically relies on binary indicators to facilitate the identification of latent classes and improve their interpretability [39]. LCA is further explained under statistical analysis.

### Reasons why non-users do not order meals for delivery

Non-users were asked to select reasons for not ordering meals for delivery from a list of responses (Table 6). For each potential reason, non-users were asked to indicate whether it was a reason to them or not (yes/no). Additionally, non-users were asked if they would be tempted to order meals for delivery if they could easily get healthy meals delivered (yes/no) or if they could easily get affordable meals delivered (yes/no) (Additional file 2).

### Statistical analysis

Socio-demographic and health characteristics were explored descriptively for the whole sample and separately for users and non-users. To identify meal delivery user profiles, an LCA was conducted using indicators related to meal delivery ordering amongst users. An LCA is a form of finite mixture modelling that identifies unobserved subgroups (latent classes) based on observed categorical variables [40]. LCA identifies mutually exclusive classes that maximise between-group differences and minimise within-group differences based on response patterns [40]. First, indicators with frequencies ≤ 15% of the sample were excluded from analysis (e.g., the importance of taste, ordering meals that match cultural or religious background). Categories with low frequencies are difficult to model due to the limited distributional information, and increase the risk of identifying a latent class solely driven by those categories, compromising the “latency” of the identified class [41]. Second, remaining indicators were checked for multicollinearity using the tetrachoric correlation matrix (Additional file 3). The pair of “order more often when dark outside” and “order more often when it rains” was highly correlated (*r* = 0.61), and therefore combined into a joint item capturing ordering more often when it is dark and/or rainy. Logit latent class models were fitted using 17 indicators related to meal delivery ordering (Table 2), with robust standard errors. As per recommended practice [40], models were run using 100 random sets of starting values with 100 iterations per set. To identify the ideal number of classes, models of 1 to 4 classes were tested and conditional independence checked. Model fit criteria were assessed across models to determine which one best represented the data. These criteria included model convergence, Bayesian information criterion (BIC), log-likelihood, class size, average maximum posterior probabilities, visual inspection of conditional probability charts, and interpretability. Average maximum posterior probabilities per latent class were calculated by averaging the maximum posterior probabilities for participants classified to a specific class. These values range from 0 to 1, with higher values indicating better homogeneity and class assignment accuracy, the most important characteristics of a good latent class model [42]. Each participant was assigned to the latent class for which they had the highest posterior probability. Resulting classes were further compared in terms of their socio-demographic and health characteristics, and frequency of meal delivery use. For non-users (i.e., both former users and never users), reasons for not ordering food for delivery were explored descriptively. Reasons for not ordering food for delivery were compared between former users and never users. Former users and never users were also compared in terms of their socio-demographic and health characteristics. Comparisons were done using Chi-Square test for categorical variables and Wilcoxon rank-sum test for non-normally distributed continuous variables.

Only participants with complete data on the indicator variables were considered in the analysis. A complete case analysis was conducted assuming data were Missing Completely At Random (MCAR). Analyses were conducted in Stata 18.

## Results

Of the 1304 participants who completed the 2024 Meal Delivery Survey, 821 (63%) were users and 483 (37%) were non-users of meal delivery services. Of the 821 users, 720 (88%) had complete data on indicators related to meal delivery use. Of the 483 non-users, 458 (95%) had complete data on variables related to reasons for not ordering meals for delivery. Amongst those non-users, 92 (20%) reported that meal delivery services were not offered where they live, those participants were excluded from analysis as the lack of service in their area is the primary reason for not ordering (e.g., one cannot claim to avoid ordering due to cost concerns without access to the service in the first place). Consequently, the sample size for the non-user analysis was *n* = 366. Sample descriptive statistics are detailed in Table 1.

**Table 1:** Descriptive statistics of whole sample and meal delivery service users vs. non-users

	Full sample (*n* = 1086)	Users (*n *= 720)	Non-user (*n* = 366)	*p*-value*
Gender				
Man	417 (39.2%)	277 (39.5%)	140 (38.8%)	0.83
Woman	646 (60.8%)	425 (60.5%)	221 (61.2%)	
Age				
Median (p25, p75)	35 (28, 51)	33 (28, 42)	46 (30, 65)	< 0.001
Ability to manage on income				
Very/difficult	96 (9.5%)	65 (9.7%)	31 (9.0%)	0.60
Just getting by	275 (27.2%)	175 (26.2%)	100 (29.2%)	
Very/comfortable	640 (63.3%)	428 (64.1%)	212 (61.8%)	
Employment status				
Employed	666 (66.1%)	487 (73.2%)	179 (52.3%)	< 0.001
Studying	99 (9.8%)	67 (10.1%)	32 (9.4%)	
Unemployed, homemaker or unable to work	103 (10.2%)	66 (9.9%)	37 (10.8%)	
Retired	139 (13.8%)	45 (6.8%)	94 (27.5%)	
Education				
Less than university	462 (46.0%)	289 (43.5%)	173 (50.7%)	0.030
University	543 (54.0%)	375 (56.5%)	168 (49.3%)	
Residential location type				
City centre	477 (44.0%)	328 (45.6%)	149 (40.7%)	0.49
Outskirts city	393 (36.2%)	252 (35.0%)	141 (38.5%)	
Village centre	140 (12.9%)	91 (12.7%)	49 (13.4%)	
Countryside or connecting road	75 (6.9%)	48 (6.7%)	27 (7.4%)	
Living situation				
Live with other people	693 (66.9%)	478 (70.6%)	215 (59.9%)	< 0.001
Live by myself	343 (33.1%)	199 (29.4%)	144 (40.1%)	
Presence of children in household				
No children	778 (75.2%)	489 (72.3%)	289 (80.7%)	0.011
At least one child ≤ 4y/o	173 (16.7%)	125 (18.5%)	48 (13.4%)	
Only children > 4y/o	83 (8.0%)	62 (9.2%)	21 (5.9%)	
Self-rated health				
Poor or fair	341 (35.7%)	242 (38.1%)	99 (30.9%)	0.072
Good	392 (41.0%)	247 (38.9%)	145 (45.3%)	
Very good or excellent	222 (23.2%)	146 (23.0%)	76 (23.8%)	
BMI				
Median (p25, p75)	24.5 (21.9, 28.2)	24.7 (22.0, 28.7)	24.0 (21.6, 27.2)	0.026

*p-values of Chi-Square tests and the Wilcoxon rank-sum tests comparing users and non-users

### Users versus non-users

Users were younger than non-users (median age: 33 vs. 46). Nearly three-quarters of users were employed, whilst only half of non-users were employed. Most users (56%) had university-level education, whilst non-users were more evenly distributed across education levels. Although most users and non-users lived with other people, a larger percentage of non-users lived alone (40%) compared to users (29%) (Table 1).

### User analysis

Meal delivery applications were the most common method for ordering food, reported by 91% of users (*n* = 659). Over half (*n* = 370, 51%) reported using restaurant websites, while phone calls were the least common, reported by 23% (*n* = 162).

Table 2 provides the descriptive statistics for the indicators related to meal delivery ordering. Fast delivery, not too costly, and tasty meals were endorsed by the large majority of participants.

**Table 2:** Frequency of endorsed indicators related to meal delivery ordering amongst users

	Users (*n* = 720)
*Time-related indicators*	
Important: delivery speed is fast	541 (75.1%)
Reason: easier to finish household chores	359 (49.9%)
Reason: provides more time for leisure activities (e.g., resting, reading, watching TV)	487 (67.6%)
*Cost-related indicators*	
Important: meal does not cost too much	569 (79.0%)
Behaviour: choose order based on promotions	368 (51.1%)
Behaviour: order more than originally planned for free delivery	433 (60.1%)
*Health-related indicators*	
Important: meals ordered are healthy	195 (27.1%)
Reason: easier to eat healthy	39 (5.4%)*
Reason: provides more time for healthy activities (e.g., exercising)	103 (14.3%)*
*Taste*	
Important: meals ordered are tasty	707 (98.2%)*
*Variety-related indicators*	
Reason: allows to try different cuisines or meals	347 (48.2%)
Behaviour: usually order from a different restaurant	190 (26.4%)
*Leftovers*	
Behaviour: deliberately order more food than needed to have leftovers	228 (31.7%)
*Culture indicator*	
Behaviour: order meals that match culture/religious background	54 (7.5%)*
*Accessibility*	
Reason: allows to order from stores that are not close by	258 (35.8%)
*Convenience-related indicators*	
Reason: allows to enjoy a (restaurant) meal when unable to go out	465 (64.6%)
Reason: avoid traffic	187 (26.0%)
Reason: avoid dining in restaurants	212 (29.4%)
Reason: avoid going to a supermarket to buy ingredients for a meal	439 (61.0%)
*Skills*	
Reason: limited/no cooking skills	108 (15.0%)*
*External conditions*	
Behaviour: order more often when it is dark and/or rainy	404 (56.1%)
*Socialising*	
Reason: easier to have social meals with friends	310 (43.1%)

“Endorsed” refers to users responding affirmatively (e.g., yes, this is important); * Indicators with frequencies ≤ 15% of the sample were excluded from analysis [41]

#### Latent class analysis

**Fig. 1:**
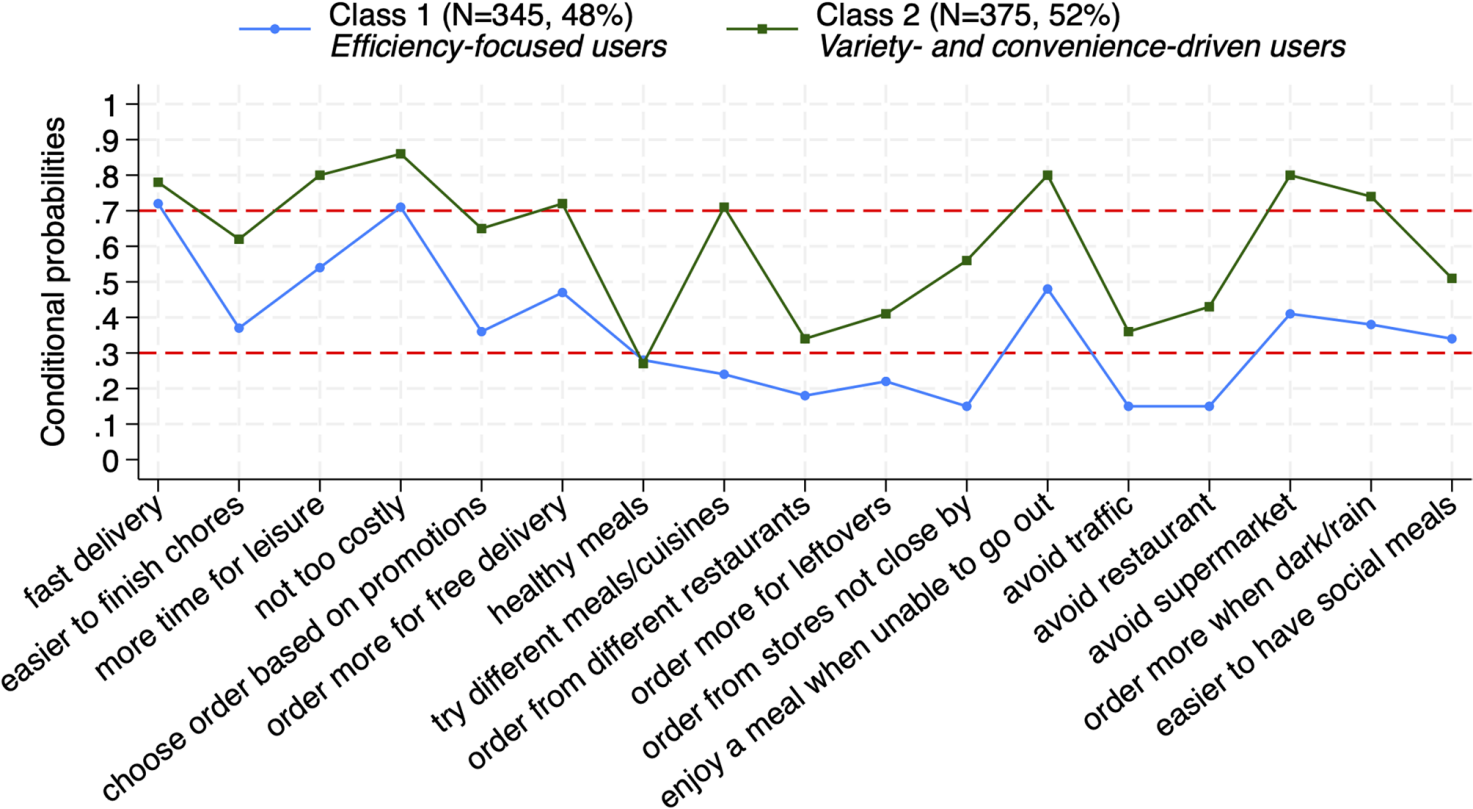
Probability of indicator endorsement conditional on class membership (only users). Note: The horizontal dashed lines provide a visual guide for high class homogeneity (i.e., estimated values greater than 0.7 and less than 0.3) and for class separation

Table 3 presents model fit criteria across the latent class models. The four-class model was not well-identified. The three-class model resulted in diminishing gains on model fit criteria (higher BIC and lower average maximum posterior probabilities) and was conditionally dependent. The two-class model demonstrated the best model fit, with the lowest BIC. The average maximum posterior probabilities for the two-class model were 0.88 for both class 1 and class 2, providing evidence of homogeneity for each class. The two-class model was therefore selected. Conditional probabilities are shown in Fig. 1. Conditional probabilities capture the probability of indicator endorsement conditional on class membership.

**Table 3:** Model fit criteria across models

Statistic	1 class	2 classes	3 classes	4 classes
LL (Log-Likelihood)	−7747.008	−7536.626	−7486.122	Model non-identification
BIC	15605.86	15303.53	15320.94	
Class 1 size (n, %)	*n* = 720, 100%	*n* = 345, 47.92%	*n* = 261, 36.25%	
Class 2 size (n, %)		*n* = 375, 52.08%	*n* = 128, 17.78%	
Class 3 size (n, %)			*n* = 331, 45.97%	
Avg. posterior prob. Class 1 (mean, SD)		0.88 (0.13)	0.78 (0.15)	
Avg. posterior prob. Class 2 (mean, SD)		0.88 (0.15)	0.80 (0.16)	
Avg. posterior prob. Class 3 (mean, SD)			0.88 (0.15)	
Local Independence		Assumption met	Assumption violated

#### Latent class description

Participants were classified into two classes. Class 1 represented 48% (*n* = 345) of the sample and class 2 represented 52% (*n* = 375). Based on their conditional probabilities (Fig. 1), class 1 was labelled “*efficiency-focused users*”, and class 2 “*variety- and convenience-driven users*”. Specifically, *efficiency-focused users* were characterised by low probability of endorsement for most indicators, capturing a group that appeared particularly less driven by variety (e.g., trying different meals/cuisines; prob.=0.24) or convenience-related factors when ordering a meal for delivery (e.g., avoiding traffic or restaurant; prob.=0.15 for both). Of all indicators, *efficiency-focused users* only had high probability of endorsement for ‘fast delivery’ (prob.=0.72) and ‘not too costly’ (prob.=0.71), reflecting a more focused group in terms of endorsed indicators related to meal delivery ordering. In contrast, *variety- and convenience-driven users* were characterised by high probability of endorsement for most indicators, with some probabilities, such as for ‘more time for leisure’ ‘enjoy a meal when unable to go out’ and ‘avoid supermarket’, reaching as high as 0.8, reflecting a strong preference for convenience and more free time.

*Efficiency-focused users* and *variety- and convenience-driven users* shared similarities. The two classes were homogeneous with respect to endorsing ‘fast delivery’ and ‘not too costly’ as important factors when ordering meal delivery, although *variety- and convenience-driven users* also had higher probabilities on ‘choose order based on promotions’ (prob.=0.65) and ‘order more for free delivery’ (prob.=0.72), which was not the case for *efficiency-focused users*. Both classes also had low probabilities of finding ‘healthy meals’ an important factor (class 1 prob.=0.28; class 2 prob.=0.27). *Efficiency-focused users* and *variety- and convenience-driven users* were, however, well separated by the indicator ‘try different meals/cuisines’, with *efficiency-focused users* having a low probability of 0.24 and *variety- and convenience-driven users* a high probability of 0.71, meaning this was a highly distinctive feature between the two classes.

#### Socio-demographic and health profiles of the efficiency-focused class and variety- and convenience-driven class

Whilst users in both classes were primarily employed, they differed in the percentage of users who were studying (*efficiency-focused*: 5% vs. *variety- and convenience-driven*: 14%) (Table 4). *Variety- and convenience-driven users* were also younger than *efficiency-focused users*. The *variety- and convenience-driven* class had more residents in city centres than the *efficiency-focused* class (50% vs. 41%) and fewer in rural areas than the *efficiency-focused* class (village centre: 10% vs. 16%; countryside or connecting road: 5% vs. 9%). When it comes to the ability to manage on income, both classes were primarily comfortable or very comfortable, although the *variety- and convenience-driven* class had more users just getting by (29% vs. 23%) or finding it difficult or very difficult to get by (11% vs. 8%) compared to the *efficiency-focused* class. Whilst both classes had mainly users living with others, there were more users living alone in the *variety- and convenience-driven* class than the *efficiency-focused* class (35% vs. 24%). In terms of self-rated health, the most common response in the *efficiency-focused* class was good health (40%), whilst poor or fair health was most frequently reported in the *variety- and convenience-driven* class (43%). The *efficiency-focused* class had more users reporting very good or excellent health than the *variety- and convenience-driven* class (27% vs. 19%). No major differences were observed in gender, education, presence of children in household and BMI between classes.

**Table 4:** Socio-demographic and health characteristics of user class 1 and user class 2

	Class 1 (*n* = 345) *Efficiency-focused users*	Class 2 (*n* = 375) *Variety- and convenience-driven users*	*p*-value*
Gender			
Man	135 (39.9%)	142 (39.0%)	0.80
Woman	203 (60.1%)	222 (61.0%)	
Age			
Median (p25, p75)	36 (30, 46)	32 (28, 38)	< 0.001
Ability to manage on income			
Very/difficult	26 (8.2%)	39 (11.1%)	0.047
Just getting by	73 (23.0%)	102 (29.1%)	
Very/comfortable	219 (68.9%)	209 (59.7%)	
Employment status			
Employed	245 (77.0%)	242 (69.7%)	< 0.001
Studying	17 (5.3%)	50 (14.4%)	
Unemployed, homemaker or unable to work	29 (9.1%)	37 (10.7%)	
Retired	27 (8.5%)	18 (5.2%)	
Education			
Less than university	148 (46.7%)	141 (40.6%)	0.12
University	169 (53.3%)	206 (59.4%)	
Residential location type			
City centre	140 (40.6%)	188 (50.3%)	0.002
Outskirts city	119 (34.5%)	133 (35.6%)	
Village centre	55 (15.9%)	36 (9.6%)	
Countryside or connecting road	31 (9.0%)	17 (4.5%)	
Living situation			
Live with other people	247 (76.2%)	231 (65.4%)	0.002
Live by myself	77 (23.8%)	122 (34.6%)	
Presence of children in household			
No children	220 (68.1%)	269 (76.2%)	0.054
At least one child ≤ 4y/o	67 (20.7%)	58 (16.4%)	
Only children > 4y/o	36 (11.1%)	26 (7.4%)	
Self-rated health			
Poor or fair	100 (32.7%)	142 (43.2%)	0.010
Good	123 (40.2%)	124 (37.7%)	
Very good or excellent	83 (27.1%)	63 (19.1%)	
BMI			
Median (p25, p75)	24.4 (21.9, 28.4)	25.0 (22.0, 29.3)	0.29

*p-values of Chi-Square tests and the Wilcoxon rank-sum tests comparing class 1 and class 2

#### Frequency of meal delivery service usage

A larger percentage of *variety- and convenience-driven users* ordered meal delivery services twice or more each month compared to *efficiency-focused users* (58% vs. 41%) (Table 5).

**Table 5:** Frequency of meal delivery service use per user latent class

	Full sample users (*n* = 720)	Class 1 (*n* = 345) *Efficiency-focused users*	Class 2 (*n* = 375) *Variety- and convenience-driven users*	*p*-value*
Frequency of use				
≤ 1/month	358 (49.7%)	202 (58.6%)	156 (41.6%)	< 0.001
≥ 2/month	362 (50.3%)	143 (41.4%)	219 (58.4%)	

*p-values of Chi-Square test comparing class 1 and class 2

### Non-user analysis

Table 6 presents the reasons for not ordering meals for delivery amongst non-users. Most non-users reported a preference for in-store food shopping (75%) and cooking their own meals (79%). Cooking own meals was the reason most often reported for both former (76%) and never users (82%). However, a higher percentage of never users (81%) reported a preference for in-store food shopping as a reason for non-use compared to former users (70%). Similarly, almost 60% of never users reported the importance of cooking together with family as a reason for non-use but this was reported for 47% of former users. Whilst lack of trust was a reason for a small minority of former users (6% due to food allergies; 13% in the hygiene of meals), 22% of never users reported lack of trust due to food allergies and almost a third (32%) did not trust the hygiene of meals. Almost a quarter of never users (24%) also thought meal delivery options are not tasty, but that was only the case for less than 15% of former users. In contrast, more than two-thirds of former users (67%) reported costs as a reason for non-use, whilst this was less than half amongst never users (44%). Thinking the food takes too long to be delivered was a reason for non-use amongst 41% of former users but only 30% of never users. No major differences were observed for the other listed reasons.

**Table 6:** Reasons why non-users do not order meals for delivery

	Full sample non-users (*n* = 366)	Former users (*n* = 190)	Never users (*n* = 176)	*p*-value*
Prefer to cook my own meals	289 (79.0%)	144 (75.8%)	145 (82.4%)	0.12
Prefer to shop for food at supermarkets	275 (75.1%)	132 (69.5%)	143 (81.2%)	0.009
Don’t want to spend money with meal delivery services	248 (67.8%)	125 (65.8%)	123 (69.9%)	0.40
Costs too much	204 (55.7%)	127 (66.8%)	77 (43.8%)	< 0.001
Cooking with family is important	192 (52.5%)	89 (46.8%)	103 (58.5%)	0.025
Prefer to eat in a restaurant	183 (50.0%)	98 (51.6%)	85 (48.3%)	0.53
Tempted to order if I could easily get affordable meals delivered	162 (44.3%)	90 (47.4%)	72 (40.9%)	0.21
Someone in my home often cooks meals I like	160 (43.7%)	80 (42.1%)	80 (45.5%)	0.52
Bad for the environment	142 (38.8%)	73 (38.4%)	69 (39.2%)	0.88
Unhealthy	136 (37.2%)	75 (39.5%)	61 (34.7%)	0.34
Long delivery time	129 (35.2%)	77 (40.5%)	52 (29.5%)	0.028
Tempted to order if I could easily get healthy meals delivered	93 (25.4%)	49 (25.8%)	44 (25.0%)	0.86
Lack trust in the hygiene of meals	82 (22.4%)	25 (13.2%)	57 (32.4%)	< 0.001
Heard negative things	80 (21.9%)	44 (23.2%)	36 (20.5%)	0.53
Not tasty	70 (19.1%)	28 (14.7%)	42 (23.9%)	0.027
Lack trust in ingredients due to food allergies	50 (13.7%)	11 (5.8%)	39 (22.2%)	< 0.001
Don’t like trying new foods	20 (5.5%)	7 (3.7%)	13 (7.4%)	0.12

Reasons are listed in order of frequency (from most to least frequently reported) amongst the full sample of non-users; *p-values of Chi-Square tests comparing former users and never users

#### Socio-demographic and health profiles of former users and never users

A higher percentage of former users were employed compared to never users (58% vs. 46%) and fewer were retired (18% vs. 38%). Former users were also younger than never users (median age: 40 vs. 57). Whilst most former users and never users lived with others, a higher percentage of never users lived alone compared to former users (46% vs. 34%). No major differences were observed between former users and never users for other socio-demographic and health characteristics. Additional file 4 presents the full table of the socio-demographic and health characteristics of former and never users.

## Discussion

This study explored characteristics of users and non-users of meal delivery services. Two latent classes of users were identified. The *efficiency-focused users* mainly endorsed indicators related to the importance of fast delivery speed as well as the order not costing too much. The *variety- and convenience-driven users* endorsed most indicators, with high probabilities on convenience, free- time, and variety-related indicators. Amongst non-users, the majority seemed to be driven by a preference for in-store food shopping and home cooking, although differences were observed in reasons for non-use between former users and those who had never used meal delivery services.

Previous research on meal delivery services has highlighted similar cost, time savings and convenience factors as primary drivers of usage [27, 43]. This study adds nuance by identifying distinct user profiles and highlighting variations in attitudinal and behavioural patterns. The *efficiency-focused users* class appeared to take a more utilitarian approach towards the use of meal delivery. A utilitarian approach to consumption can be understood as instrumental, oriented towards achieving a specific purpose and based on how useful the purchased good or service is [44, 45]. These utility-driven users tend to order more occasionally, likely out of situational necessity. Importantly, their use appeared to be primarily functional, with decisions based on clear criteria (speed and cost) rather than being influenced by promotions or non-essential features. The *efficiency-focused users* label intended to capture this practical approach to meal delivery. In contrast, *variety- and convenience-driven users* tend to be more frequent users who value meal delivery services for the convenience they offer and the leisure opportunities they create, such as avoiding grocery shopping or freeing up time for other activities. This group also appeared interested in the opportunity to try different meals or cuisines and driven by additional offers and discounts.

No geographical differences were observed between users and non-users, suggesting that although urban areas likely provide greater access to meal delivery services [46], accessibility alone is not a sufficient driver for meal delivery usage. Previous research showed that most online shoppers in Belgium are located in dense urban areas, although this applied to general online shopping not specifically ready-to-eat meals [47]. When looking at user classes, we did observe a geographical pattern, with more users from city centres and fewer from rural areas for *variety- and convenience-driven users* compared to *efficiency-focused users*. This may reflect the wider choice of restaurants, cuisines, and promotions in cities, making meal delivery particularly appealing for *variety- and convenience-driven users*. In rural areas, fewer options and potentially longer delivery times may mean users focus more on efficiency. These differences in user classes support the innovation-diffusion hypothesis for online shopping adoption [48, 49], which suggests that new technologies start in these centres of innovation (i.e., urban centres) before moving to other areas over time. The widespread adoption of meal delivery services is still a relatively recent phenomenon in Belgium.

The fact that both classes valued a speedy delivery and affordability suggests that these are universally important when ordering food for delivery. Time efficiency and affordability have been recognised as primary factors when ordering non-food items for delivery [28, 50, 51]. Meal delivery services are no different, users value getting meals at a reasonable price and receiving them as quickly as possible. Both classes also found the healthfulness of the meals unimportant when using meal delivery services. This finding aligns with previous research indicating that choosing an out-of-home-food outlet is rarely primarily done for health reasons [52]. Short-term goals, such as eating a quick, low-effort, and tasty meal, often outweigh long-term health goals, even amongst health-conscious individuals [52]. Similarly, qualitative research has suggested consumers do not use meal delivery services with the intent of purchasing healthy food [53]. The heavy marketing of unhealthy food for delivery may also reinforce the perception that meal delivery is mainly for indulgence rather than health [54, 55]. If unhealthy options dominate advertisements and promotions, consumers may associate meal delivery with unhealthy meals and not consider it a source of healthier meals. This could shape default ordering behaviours, making unhealthy choices the norm when ordering food for delivery. To encourage healthier food choices, consumer perceptions may need to shift by making healthier options more visible, accessible, and appealing for delivery. For example, advertisements for meal delivery platforms could show healthier items instead of typical options like pizzas and burgers. Within the platforms themselves, healthier meals could be promoted at the top of lists, with appealing photos and descriptions. Menus could also include nutritional information, helping users make more informed decisions amongst the large majority of unhealthy options. However, such measures may only benefit a minority of health-conscious consumers [56, 57]. Another consideration to encourage healthier food choices may be on the supply side, such as increasing the number of healthy items or the number of healthy food stores offering delivery. However, barriers for food businesses should be acknowledged. Some healthier meals (e.g., soups, salads) may be harder to deliver in optimal condition, especially given the current delivery infrastructure (e.g., the reliance on bike couriers carrying food in backpacks). Owners and managers of (healthy) food businesses may be reluctant to offer meal delivery as they are concerned about losing control over meal quality [58] (e.g., risk of spilling, crushing or sogginess). Another related consideration may be to improve the healthfulness of existing options and make currently unhealthy meals more nutritious. It should, however, be noted that assumptions about the unhealthfulness of food available for delivery are mainly based on international evidence [6–8]. The availability and healthfulness of options in the Belgian context remain unexplored. Opportunities exist to quantify the availability of healthy options on meal delivery platforms in Belgium.

Our findings related to age differences align with previous research suggesting users of meal delivery services are typically younger [32, 33], which may be explained by the fact that younger adults are more likely to engage with mobile technology and more interested in food exploration than older adults [23]. Non-users, who were characterised by older age, especially those who had never used meal delivery services, reported preferring to shop for food in stores and cook their own meals as reasons for not using meal delivery services. Previous research has suggested that older adults tend to eat home-cooked meals more frequently [59, 60], and allocate a larger proportion of their food budget to fresh and non-convenient meal ingredients [61]. These findings may represent generational differences in food consumption and time availability. Older individuals are less in the habit of consuming convenient meals [61], more resistant to change in established (eating) routines, and often retired, leaving more free time to prepare home-cooked meals [62]. It is worth noting that many workers in Belgium retire (i.e., receive old-age pension benefits) before the age of 65 years [63], which may explain the lower number of participants over 65 years old, whilst 14% of the sample were retired.

A larger proportion of users were employed compared to non-users. However, no differences were observed in the ability to manage on income between users and non-users. Thus, it is possible that those who are employed experience greater time constraints than those who are not [64]. Previous research has shown that work-related time demands may encourage out-of-home food purchasing [65, 66] as a means to cope with limited time to visit outlets, purchase and cook food at home [67]. With meals brought directly to them, workers may be even more inclined to resort to meal delivery services. However, research is yet to explore the specific impact of work-related time scarcity on the use of meal delivery services.

We observed differences in the self-rated health of user profiles, but not in BMI. The *variety- and convenience-driven* class, who included more frequent users of meal delivery services, had poorer self-rated health compared to the *efficiency-focused* class, who included fewer frequent users. It might be that more frequent use of meal delivery services means poorer overall diet quality [7, 33], which has been linked to poorer quality-of-life, health and wellbeing [68], impacting perceived health. An alternative explanation may be that *variety- and convenience-driven users* value being healthy less, thus make more unhealthy choices, including more meal delivery ordering. However, the nutritional quality of the diets of meal delivery users remains largely unexplored. It is also unclear whether individuals who frequently order unhealthy meals for delivery compensate with a healthier diet overall. In our sample, *efficiency-focused users* consumed a daily average of 1.46 servings of fruit and 2.25 servings of vegetables, whilst *variety- and convenience-driven users* consumed a daily average of 1.28 servings of fruit and 2.27 servings of vegetables (results not shown). Future research should explore the dietary and health implications of meal delivery service use, including potential compensatory behaviour.

Given the growth of meal delivery services [1, 2], understanding their impact on food behaviours is important to ensure adequate policy responses. If meal delivery mainly replaces dining out or picking up takeaway, it may not necessarily increase the health burden from out-of-home food. It would simply represent a shift in how out-of-home food is accessed and consumed rather than an increase in its consumption (although it would still warrant public health attention due to the negative health implications of such food [5]). The implications may be more concerning if meal delivery services add to, rather than replace, existing out-of-home food consumption. Research suggests ordering meals for delivery may complement dining in restaurants but replace travelling for takeaway meals [29]. However, early findings around the substitutive or complementary effect of ordering in remain inconsistent [30], warranting more research. Previous seminal work focusing on online retailing more broadly had explored potential implications of multi-channel retailing, raising important questions about, e.g., how time saved by switching from conventional in-person shopping to online shopping may be reallocated to other activities [49]. These considerations are highly relevant to meal delivery usage, as getting food delivered may free up time for other behaviours. In our sample, 68% of users reported that more leisure time was an important reason for using meal delivery services. Our qualitative work is exploring this potential reallocation of time.

The study has several strengths and limitations. The survey was available in multiple languages (English, Dutch and French), allowing it to reach a large and more diverse population in the Flanders and Brussels regions of Belgium. The study was the first to explore such a wide array of indicators related to meal delivery service use and to consider reasons for non-use amongst former users and never users. Whilst we were able to capture a range of potential factors playing a role in the use of meal delivery services and potential reasons why individuals choose not to use such services, data were captured using binary responses based on the needs and interpretability of LCA. Further work is needed to unpack responses and understand the relative importance of each factor. In the present study, we captured users and non-users, with two different sets of questions to understand reasons for use and non-use. What remains unexplored here is whether (and why) never users intend to start using these services in the future and whether (and why) former users have actively decided to stop using them or may do so again in the future. In-depth qualitative work that we have recently conducted is exploring such questions. We used a 6-month cut-off point to distinguish users from non-users and capture recent meal ordering behaviours. Amongst users, almost one-third reported using meal delivery services less than once a month in the past 6 months. However, it is possible that very occasional users (e.g., those who order only a couple of times a year but had not ordered in the 6 months prior to the survey) may have been classified as non-users. Future research could explore these less frequent behaviours to better understand occasional or irregular use patterns. Our sample also appeared to have a higher socio-economic status (SES) than the general population. Future research should investigate meal delivery ordering behaviours of lower SES groups to better understand potential differences and implications for inequalities. Respondents had a median age of 35 years (25th percentile: 28; 75th percentile: 51). More specifically, users had a median age of 33 years (28; 42), whilst non-users were older, with a median age of 46 years (30; 65). This age pattern appears to be reflective of individuals with an online presence and typical age ranges associated with both the use (younger) and non-use (older) of meal delivery services. Some methodological limitations should also be noted. We used LCA to identify latent subgroups of users based on a set of characteristics. As a probability-based method, LCA cannot definitively assign an individual to a class. Assignment of users to latent classes was based on their highest posterior probability [40, 42]. These latent patterns should not be considered as exact attitudes and behaviours but rather as representations of more complex attitudinal and behavioural patterns.

## Conclusion

This study characterised profiles of meal delivery service users and non-users in Belgium. Users fell into two groups: *efficiency-focused users*, who prioritised fast and affordable service, and *variety- and convenience-driven users*, who valued a wider range of factors, including convenience, free time and variety. The latter group included more frequent users. Amongst non-users, both *former users* and *never users* reported various reasons for not using these services, with preferences for in-store food shopping and home cooking being the most frequently reported. Differences in socio-demographic and health characteristics were observed across profiles. Given the growth of meal delivery services and their potential health impacts, these findings provide timely and warranted insights into who uses meal delivery services and why, informing public health strategies aimed at improving food behaviours.

## Supplementary Information

Additional file 1. Flowchart of inclusion and exclusion of respondents.

Additional file 2. Survey questions.

Additional file 3. Tetrachoric correlation matrix of indicators related to meal delivery service use.

Additional file 4. Socio-demographic and health characteristics of former and never users.

## Data Availability

The dataset analysed in this current study is available from the corresponding author on reasonable request.

## Abbreviations

BIC: Bayesian information criterion
BMI: Body mass index
FWO: Fonds Wetenschappelijk Onderzoek
LCA: Latent class analysis
SES: Socio-economic status
