# Supplementary figures and images for: Profiling users and non-users of meal delivery services in Belgium using latent class analysis
Source: Int J Behav Nutr Phys Act. 2025 Oct 30;22:133. doi: 10.1186/s12966-025-01827-3 (PMC12577361; doi:10.1186/s12966-025-01827-3)

**Additional file 1: Flowchart of inclusion and exclusion of respondents**


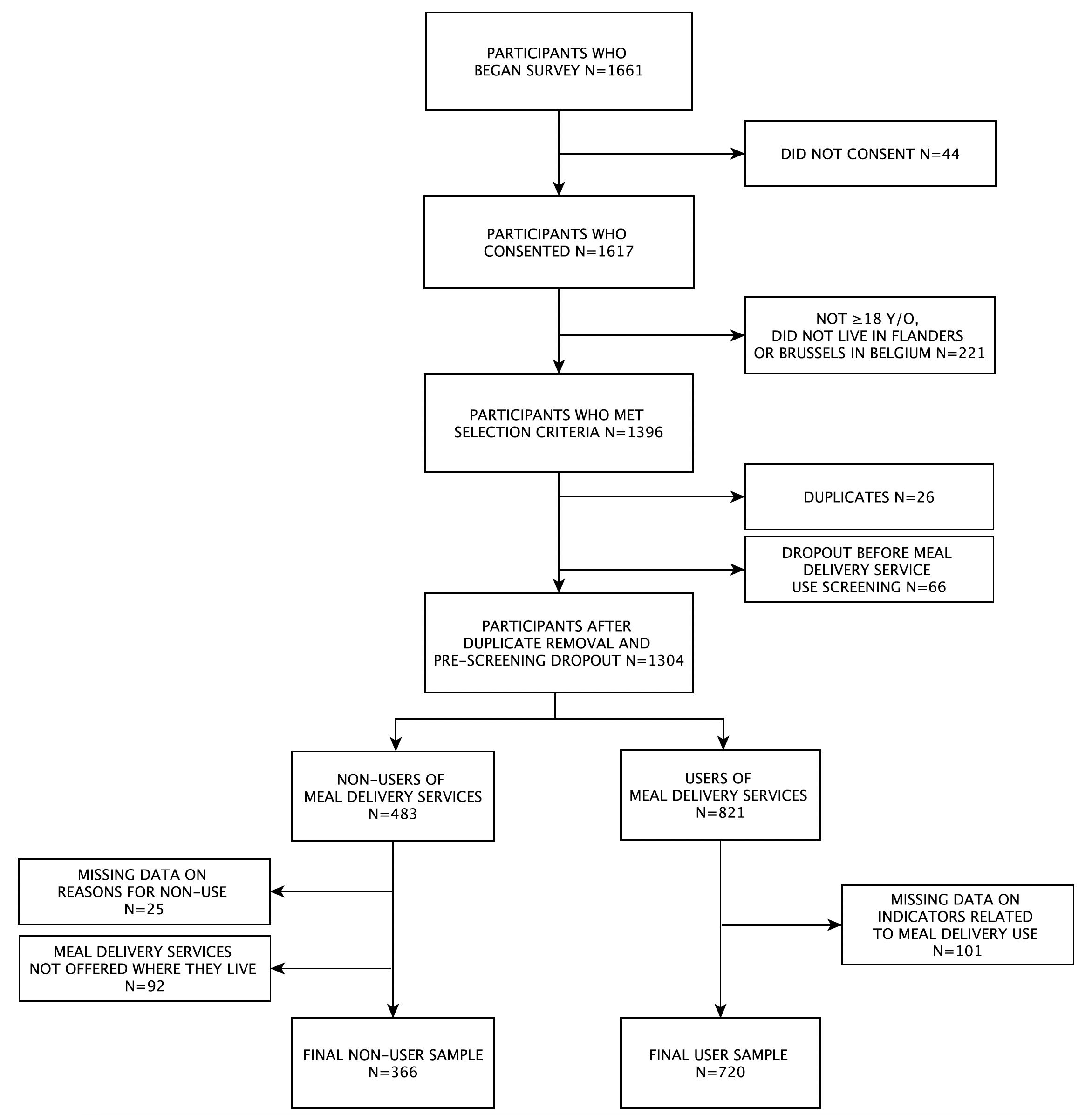

Supplement: Supplementary file 1 — Additional file 1. Flowchart of inclusion and exclusion of respondents. [file 12966_2025_1827_MOESM1_ESM.docx]
