# Supplementary material for: Profiling users and non-users of meal delivery services in Belgium using latent class analysis
Source: Int J Behav Nutr Phys Act. 2025 Oct 30;22:133. doi: 10.1186/s12966-025-01827-3 (PMC12577361; doi:10.1186/s12966-025-01827-3)
Supplement: Supplementary file 2 — Additional file 2. Survey questions. [file 12966_2025_1827_MOESM2_ESM.docx]

**Additional file 2: Survey questions**

| **Variable** | **Question in Meal Delivery Survey** | **Categories** |
| --- | --- | --- |
| **Gender** | What is your gender? | 1 Man 2 Woman 3 Other |
| **Age** | What year were you born in? | Year |
| **Ability to manage on income** | Based on your current income and expenses, how would you rate your ability to manage financially? E.g., paying for bills, food or school expenses | 1 Very difficult 2 Difficult 3 Just getting by 4 Comfortable 5 Very comfortable |
| **Education** | What is the highest qualification you have completed? | 1 No diploma 2 Lower education 3 Lower secondary education of the 1st or 2nd degree 4 Higher secondary education or secondary education of the 3rd degree 5 Post-secondary not-higher education (4th grade 7th year, training management  small enterprises ,...) 6 Higher education outside the university - short type, graduate (A1), professional bachelor 7 Higher education outside the university - the long type, master at a high school 8 Academic bachelor (higher school or university) 9 University, licentiate,  engineer, master, or doctorate with thesis |
| **Employment status** | In a usual week, which of the following best describes your primary activity and/or responsibility? | 1 Employed 2 Homemaker or caring for family 3 Studying 4 Unemployed 5 Long-term sick-leave/unable to work 6 Retired |
| **Residential location type** | Do you consider your residential location to be: | 1 city centre 2 outskirts city 3 village centre 4 countryside or connecting road |
| **Living situation** | Do you live alone? | 0 Live with other people 1 Live by myself |
| **Household composition** | Not including yourself, how many people live in your household most nights of the week? | 0 0 people 1 1 person 2 2 people 3 3 people 4 4 people 5 5 or more people |
|  | Children (4 years or younger) |  |
|  | Children (5 to 12 years) |  |
|  | Children (13 to 17 years) |  |
|  | Adults (18 to 25 years) |  |
|  | Adults (26 to 45 years) |  |
|  | Adults (46 to 65 years) |  |
|  | Adults (66 years or older) |  |
| **Self-rated health** | In general, how would you rate your health? | 1 Poor 2 Fair 3 Good 4 Very good 5 Excellent |
| **Height (cm)** | How tall are you without your shoes? Answer in centimetres (cm). | Centimetres |
| **Weight (kg)** | How much do you weigh without clothes or shoes? Answer in kilograms (kg). | Kilograms |
| **Ordered meal delivery: ever** | Have you ever had a ready-to-eat meal delivered to your home?  For example, pizza or sushi ordered through online apps such UberEats, Takeaway.com, or directly from a restaurant website, or via phone call to a restaurant. | 0 No 1 Yes |
| **Ordered meal delivery: in the last 6 months** | Have you had a ready-to-eat meal delivered to your home in the last 6 months? | 0 No 1 Yes |
| **Frequency of meal delivery** | In a normal month, how often do you order a meal for delivery? | 1 <1/month 2 1/month 3 2/month 4 3/month 5 4/month 6 ≥5/month |
| **Ordering method** | Which of the following methods do you use when ordering meals for delivery? |  |
| Method: meal delivery apps | Meal delivery apps | 0 No 1 Yes |
| Method: restaurant website | Restaurant website | 0 No 1 Yes |
| Method: phone call | Phone | 0 No 1 Yes |
| **Important** | When ordering meals for delivery, which of the following factors are important to you? Please report whether each factor is "Important to me" or "Not important to me". |  |
| Important: does not cost too much | It does not cost too much. | 0 Not important to me 1 Important to me |
| Important: fast | Delivery speed is fast. | 0 Not important to me 1 Important to me |
| Important: tasty | Meals ordered are tasty. | 0 Not important to me 1 Important to me |
| Important: healthy | Meals ordered are healthy. | 0 Not important to me 1 Important to me |
| **Reason for ordering** | Based on your experiences and preferences, please indicate whether the following are reasons you order food for delivery. For each reason, choose either 'Yes, that is a reason why I order food for delivery' OR 'No, that is not a reason why I order food for delivery'. |  |
| Reason for ordering: easier to finish household chores | It makes it easier for me to finish other household chores. | 0 No, not a reason 1 Yes, a reason |
| Reason for ordering: easier to eat healthy | It makes it easier for me to eat healthy. | 0 No, not a reason 1 Yes, a reason |
| Reason for ordering: easier to have social meals | It makes it easier for me to have social meals with friends. | 0 No, not a reason 1 Yes, a reason |
| Reason for ordering: allows to order from stores not close by | It allows me to order from stores that are not close to where I live. | 0 No, not a reason 1 Yes, a reason |
| Reason for ordering: allows to try different cuisines/meals | It allows me to try different cuisines or meals. | 0 No, not a reason 1 Yes, a reason |
| Reason for ordering: allows to enjoy a (restaurant) meal when unable to go out | It allows me to enjoy a (restaurant) meal when I am unable to go out. | 0 No, not a reason 1 Yes, a reason |
| Reason for ordering: time for healthy activities | It gives me more free time for healthy activities such as exercise. | 0 No, not a reason 1 Yes, a reason |
| Reason for ordering: time for leisure activities | It gives me more free time for other activities such as resting, reading, watching TV, gaming. | 0 No, not a reason 1 Yes, a reason |
| Reason for ordering: traffic avoidance | I use meal delivery services to avoid traffic. | 0 No, not a reason 1 Yes, a reason |
| Reason for ordering: restaurant avoidance | I use meal delivery services to avoid dining in restaurants | 0 No, not a reason 1 Yes, a reason |
| Reason for ordering: supermarket avoidance | I use meal delivery services to avoid going to a supermarket to buy ingredients for a meal. | 0 No, not a reason 1 Yes, a reason |
| Reason for ordering: limited/no cooking skills | I have limited/no cooking skills. | 0 No, not a reason 1 Yes, a reason |
| **Behaviour** | Based on your experiences and preferences, please indicate whether you engage in the following behaviours related to meal delivery. |  |
| Behaviour: order more when dark outside | I order meal delivery more often when it is dark outside. | 0 No, I don’t 1 Yes, I do |
| Behaviour: order more when it rains | I order meal delivery more often when it rains. | 0 No, I don’t 1 Yes, I do |
| Behaviour: order from different restaurant | I usually order from a different restaurant every time I order meal delivery. | 0 No, I don’t 1 Yes, I do |
| Behaviour: order more for leftovers | I deliberately order more food than needed so that I have leftovers. | 0 No, I don’t 1 Yes, I do |
| Behaviour: choose order based on promotions | I choose my order (i.e., the restaurant and/or the meal itself) based on promotions that mean better value for money. | 0 No, I don’t 1 Yes, I do |
| Behaviour: order more for free delivery | I order more than originally planned to meet minimum price for free delivery. | 0 No, I don’t 1 Yes, I do |
| Behaviour: order match culture/religious background | I order meals that match my cultural/religious background. | 0 No, I don’t 1 Yes, I do |
| **Reason for not ordering** | Please indicate whether the following are reasons you do not order food for delivery. For each reason, choose either 'Yes, that is a reason why I don't order' OR 'No, that is not a reason why I don't order.' |  |
| Reason for not ordering: costs too much | I think it costs too much. | 0 No, not a reason 1 Yes, a reason |
| Reason for not ordering: unhealthy | I think meal delivery options are unhealthy. | 0 No, not a reason 1 Yes, a reason |
| Reason for not ordering: not tasty | I think meal delivery options are not tasty. | 0 No, not a reason 1 Yes, a reason |
| Reason for not ordering: bad for the environment | I think meal delivery services are bad for the environment. | 0 No, not a reason 1 Yes, a reason |
| Reason for not ordering: don't like trying new foods | I don’t like trying different foods. | 0 No, not a reason 1 Yes, a reason |
| Reason for not ordering: don't want to spend money with meal delivery services | I don’t want to spend money with meal delivery services. | 0 No, not a reason 1 Yes, a reason |
| Reason for not ordering: lack trust - food allergies | I lack trust in ingredients due to food allergies. | 0 No, not a reason 1 Yes, a reason |
| Reason for not ordering: lack trust - hygiene | I lack trust in the hygiene of meals. | 0 No, not a reason 1 Yes, a reason |
| Reason for not ordering: heard negative things | I have heard negative things about food delivery. | 0 No, not a reason 1 Yes, a reason |
| Reason for not ordering: long delivery time | I think the food takes too long to be delivered. | 0 No, not a reason 1 Yes, a reason |
| Reason for not ordering: not offered where I live | Meal delivery services are not offered where I live. | 0 No, not a reason 1 Yes, a reason |
| Reason for not ordering: cooking with family is important | I believe cooking together with my family is important. | 0 No, not a reason 1 Yes, a reason |
| Reason for not ordering: prefer to cook my own meals | I prefer to cook my own meals. | 0 No, not a reason 1 Yes, a reason |
| Reason for not ordering: prefer to eat in a restaurant | I prefer to eat in a restaurant. | 0 No, not a reason 1 Yes, a reason |
| Reason for not ordering: prefer to shop for food | I prefer to shop for food at supermarkets, markets, the local butcher's, etc. | 0 No, not a reason 1 Yes, a reason |
| Reason for not ordering: someone in my home often cooks meals I like | Someone in my home often cooks meals I like. | 0 No, not a reason 1 Yes, a reason |
| Order if I could easily get healthy meals delivered | If you could easily get healthy meals delivered, would you be tempted to order meals for delivery? | 0 No, not a reason 1 Yes, a reason |
| Order if I could easily get affordable meals delivered | If you could easily get affordable meals delivered, would you be tempted to order meals for delivery? | 0 No, not a reason 1 Yes, a reason |
