# Supplementary material for: Profiling users and non-users of meal delivery services in Belgium using latent class analysis
Source: Int J Behav Nutr Phys Act. 2025 Oct 30;22:133. doi: 10.1186/s12966-025-01827-3 (PMC12577361; doi:10.1186/s12966-025-01827-3)
Supplement: Supplementary file 3 — Additional file 3. Tetrachoric correlation matrix of indicators related to meal delivery service use. [file 12966_2025_1827_MOESM3_ESM.docx]

**Additional file 3: Tetrachoric correlation matrix of indicators related to meal delivery service use**

|  | t_fast | t_chores | t_leisure | c_nocost | c_promo | c_freedel | h_healthy | v_trydiff | v_diffrest | m_leftovers | con_notnear | con_unable | con_avoidtraf | con_avoidrest | con_avoidsup | con_dark | con_rain | con_dark_rain | s_meals |
| --- | --- | --- | --- | --- | --- | --- | --- | --- | --- | --- | --- | --- | --- | --- | --- | --- | --- | --- | --- |
|  |  |  |  |  |  |  |  |  |  |  |  |  |  |  |  |  |  |  |  |
| t_fast | 1.00 |  |  |  |  |  |  |  |  |  |  |  |  |  |  |  |  |  |  |
| t_chores | 0.16 | 1.00 |  |  |  |  |  |  |  |  |  |  |  |  |  |  |  |  |  |
| t_leisure | 0.14 | 0.38 | 1.00 |  |  |  |  |  |  |  |  |  |  |  |  |  |  |  |  |
| c_nocost | 0.01 | 0.08 | 0.11 | 1.00 |  |  |  |  |  |  |  |  |  |  |  |  |  |  |  |
| c_promo | 0.03 | 0.17 | 0.16 | 0.45 | 1.00 |  |  |  |  |  |  |  |  |  |  |  |  |  |  |
| c_freedel | 0.06 | 0.20 | 0.23 | 0.28 | 0.31 | 1.00 |  |  |  |  |  |  |  |  |  |  |  |  |  |
| h_healthy | 0.06 | 0.17 | -0.20 | -0.03 | -0.01 | -0.14 | 1.00 |  |  |  |  |  |  |  |  |  |  |  |  |
| v_trydiff | -0.06 | 0.14 | 0.13 | 0.11 | 0.17 | 0.16 | 0.16 | 1.00 |  |  |  |  |  |  |  |  |  |  |  |
| v_diffrest | 0.18 | 0.03 | 0.01 | -0.09 | 0.09 | 0.07 | 0.08 | 0.33 | 1.00 |  |  |  |  |  |  |  |  |  |  |
| m_leftovers | -0.04 | 0.11 | -0.02 | 0.09 | 0.10 | 0.32 | 0.05 | 0.20 | 0.06 | 1.00 |  |  |  |  |  |  |  |  |  |
| con_notnear | 0.03 | 0.09 | 0.15 | 0.13 | 0.21 | 0.12 | 0.08 | 0.50 | 0.12 | 0.17 | 1.00 |  |  |  |  |  |  |  |  |
| con_unable | 0.07 | 0.09 | 0.09 | 0.01 | 0.09 | 0.03 | 0.08 | 0.38 | 0.07 | 0.09 | 0.34 | 1.00 |  |  |  |  |  |  |  |
| con_avoidtraf | 0.10 | 0.21 | 0.18 | -0.10 | -0.01 | -0.06 | 0.03 | 0.15 | 0.09 | 0.09 | 0.22 | 0.30 | 1.00 |  |  |  |  |  |  |
| con_avoidrest | -0.02 | 0.05 | 0.07 | 0.09 | 0.08 | 0.04 | 0.03 | 0.24 | 0.05 | 0.18 | 0.23 | 0.39 | 0.33 | 1.00 |  |  |  |  |  |
| con_avoidsup | 0.17 | 0.26 | 0.40 | 0.04 | 0.26 | 0.20 | -0.07 | 0.22 | 0.18 | 0.16 | 0.17 | 0.22 | 0.18 | 0.36 | 1.00 |  |  |  |  |
| con_dark | 0.16 | 0.06 | 0.18 | 0.15 | 0.17 | 0.14 | -0.11 | 0.13 | 0.07 | 0.12 | 0.20 | 0.16 | 0.00 | 0.12 | 0.29 | 1.00 |  |  |  |
| con_rain | 0.27 | 0.13 | 0.27 | 0.05 | 0.21 | 0.16 | -0.11 | 0.15 | 0.12 | 0.12 | 0.21 | 0.19 | 0.20 | 0.11 | 0.25 | **0.61** | 1.00 |  |  |
| con_dark_rain | 0.17 | 0.07 | 0.28 | 0.12 | 0.21 | 0.15 | -0.16 | 0.20 | 0.11 | 0.11 | 0.28 | 0.21 | 0.07 | 0.12 | 0.28 | 1.00 | 1.00 | 1.00 |  |
| s_meals | 0.07 | -0.09 | 0.03 | -0.01 | 0.08 | 0.10 | -0.12 | 0.16 | 0.03 | -0.01 | 0.13 | 0.15 | 0.08 | 0.06 | 0.17 | 0.15 | 0.18 | 0.19 | 1.00 |
