# Supplementary material for: Profiling users and non-users of meal delivery services in Belgium using latent class analysis
Source: Int J Behav Nutr Phys Act. 2025 Oct 30;22:133. doi: 10.1186/s12966-025-01827-3 (PMC12577361; doi:10.1186/s12966-025-01827-3)
Supplement: Supplementary file 4 — Additional file 4. Socio-demographic and health characteristics of former and never users. [file 12966_2025_1827_MOESM4_ESM.docx]

**Additional file 4: Socio-demographic and health characteristics of former and never users**

|  | **Former user**  **(N=190)** | **Never user**  **(N=176)** | **p-value*** |
| --- | --- | --- | --- |
| **Gender** | | | |
| Man | 71 (38.0%) | 69 (39.7%) | 0.74 |
| Woman | 116 (62.0%) | 105 (60.3%) |  |
| **Age**, median  (p25, p75) | 40  (30, 59) | 57  (31, 68) | 0.002 |
| **Ability to manage on income** | | | |
| Very/difficult | 19 (10.5%) | 12 (7.4%) | 0.47 |
| Just getting by | 49 (27.1%) | 51 (31.5%) |  |
| Very/comfortable | 113 (62.4%) | 99 (61.1%) |  |
| **Employment status** | | | |
| Employed | 104 (57.8%) | 75 (46.3%) | <0.001 |
| Studying | 17 (9.4%) | 15 (9.3%) |  |
| Not employed | 26 (14.4%) | 11 (6.8%) |  |
| Retired | 33 (18.3%) | 61 (37.7%) |  |
| **Education** | | | |
| Less than university | 88 (48.9%) | 85 (52.8%) | 0.47 |
| University | 92 (51.1%) | 76 (47.2%) |  |
| **Residential location type** | | | |
| city centre | 71 (37.4%) | 78 (44.3%) | 0.31 |
| outskirts city | 82 (43.2%) | 59 (33.5%) |  |
| village centre | 24 (12.6%) | 25 (14.2%) |  |
| countryside or connecting road | 13 (6.8%) | 14 (8.0%) |  |
| **Living situation** | | | |
| Live with other people | 122 (65.6%) | 93 (53.8%) | 0.022 |
| Live by myself | 64 (34.4%) | 80 (46.2%) |  |
| **Presence of children in household** | | | |
| No children | 144 (77.8%) | 145 (83.8%) | 0.15 |
| At least one child ≤4y/o | 26 (14.1%) | 22 (12.7%) |  |
| Only children >4y/o | 15 (8.1%) | 6 (3.5%) |  |
| **Self-rated health** | | | |
| Poor or fair | 52 (30.6%) | 47 (31.3%) | 0.17 |
| Good | 84 (49.4%) | 61 (40.7%) |  |
| Very good or excellent | 34 (20.0%) | 42 (28.0%) |  |
| **BMI**, median  (p25, p75) | 23.8  (21.5, 26.8) | 24.1  (21.6, 27.7) | 0.55 |

*p-values of Chi-Square tests and the Wilcoxon rank-sum test comparing former users to never users
